# Transcriptome analysis identifies EDA1 variants disrupt FOSB-mediated regulation of odontogenic epithelial cell behaviors during dental germ development

**DOI:** 10.3389/fcell.2025.1701546

**Published:** 2026-01-07

**Authors:** Jing Zhang, Xuanting Kong, Yunyun Yuan, Ya Zhao, Yulin Ding, Jiabao Ren, Wenjing Shen

**Affiliations:** 1 Department of Prosthodontics, Hebei Key Laboratory of Stomatology, Hebei Technology Innovation Center of Oral Health, School and Hospital of Stomatology, Hebei Medical University, Shijiazhuang, China; 2 Department of Prosthodontics, Hebei Eye Hospital, Xingtai, China; 3 Department of Stomatology, The Second Hospital of Shijiazhuang, Shijiazhuang, China; 4 Department of Stomatology, The No. 2 Hospital of Baoding, Baoding, China; 5 Hebei Key Laboratory of Stomatology, Hebei Technology Innovation Center of Oral Health, School and Hospital of Stomatology, Hebei Medical University, Shijiazhuang, China

**Keywords:** tooth agenesis, ectodysplasin Al, cell behavior, FosB, RNAscope

## Abstract

Here, we systematically investigated the effects of EDA1 variants on apoptosis, migration, and adhesion in ameloblast-like LS8 cells and identified downstream effectors of the EDA-NF-κB pathway through RNA sequencing (RNA-seq) combined with *in vivo* and *in vitro* validation. LS8 cells were transiently transfected with four constructs: wild-type (Wt) EDA1, nonsyndromic tooth agenesis-associated EDA1 variant (EDA1-A259E), X-linked hypohidrotic ectodermal dysplasia-associated EDA1 variant (EDA1-H252L), or empty vector control (pCMV-C-FLAG). We used flow cytometry, wound-healing assay, and cell counting kit 8 assay to assess cell apoptosis, migration, and adhesion, respectively. High-throughput RNA-seq was used to identify differentially expressed genes, which were subsequently validated through quantitative polymerase chain reaction and immunoblotting. Spatiotemporal *Fosb* expression patterns were comparatively analyzed in Wt and *Tabby* mouse tooth germs through *in situ* hybridization by using RNAscope. Wt EDA1 transiently enhances cellular migratory capacity, a function compromised in the pathogenic EDA1 variants. The EDA-NF-κB pathway operates through FOSB-mediated transcriptional regulation, as evidenced by coordinated suppression of *Fosb* expression at transcriptional and translational levels in the mutant models. Therefore, FOSB is a candidate downstream effector in EDA1-mediated odontogenesis. These results provide mechanistic insights into ectodermal dysplasia pathogenesis.

## Introduction

Tooth formation is a complex developmental process involving reciprocal epithelial–mesenchymal interactions and inductive signaling cascades (Mustonen et al., 2004; Hemeryck et al., 2022). It is precisely regulated via intricate molecular signaling networks coordinating spatiotemporal cellular responses. Perturbations at any nodal point within these regulatory circuits result in dental developmental anomalies, ranging from enamel hypoplasia to tooth agenesis (TA) (Chow et al., 2024). Based on the cooccurrence of developmental anomalies in other ectodermal derivatives, congenital TA is etiologically classified into (Mustonen et al., 2004) syndromic TA (STA), characterized by multiorgan involvement, and (Hemeryck et al., 2022) nonsyndromic TA (NSTA), manifesting as isolated dental defects (Zhao et al., 2023). Current Online Mendelian Inheritance in Man records document 92 TA-associated genes (https://omim.org/, accessed 7 June 2025). Genes such as *PAX9*, *WNT10A*, *MSX1*, *AXIN2*, and *EDA* demonstrate pleiotropic capacity to induce both STA and NSTA phenotypes (Borges et al., 2025; Lan et al., 2022; Ahmed et al., 2021); of them, *EDA* has been one of the most intensively studied (Schneider et al., 2023; Yu et al., 2024).

The *EDA* (i.e., the ectodysplasin A gene), mapped to the chromosomal locus Xq12-q13.1, encodes a pivotal morphogenetic regulator belonging to the tumor necrosis factor (TNF) superfamily. Its 425-kilobase genomic locus comprises 12 exons generating two principal isoforms: EDA1 and EDA1 (Yu et al., 2023; Cai et al., 2021). EDA1 exhibits stringent receptor specificity, exclusively binding its cognate receptor EDAR to activate the canonical NF-κB signaling cascade. This molecular interaction orchestrates transcriptional regulation of downstream effector genes critical for tooth and skin appendage development (Wu et al., 2020). The *Tabby* (*Eda* ^−/−^), downless (*Edar* ^−/−^), crinkled (*Edaradd* ^−/−^) murine models exhibit identical phenotypic profiles characterized by defective pilosebaceous unit development, TA or malformation, and hypofunctional exocrine glands. This phenotypic convergence demonstrates that the functional roles of core EDA signaling components (i.e., EDA, EDAR, and EDARADD) are conserved in odontogenesis, tooth germ initiation, morphogenetic patterning, and ameloblastic differentiation (Reinartz et al., 2023). Patients harboring distinct genetic mutations leading to congenital dental hypoplasia typically present with graded tapering of the maxillary central and lateral incisors. A quantitative morphometric analysis revealed the highest tapering severity in maxillary anterior teeth among *EDA* variant carriers (Ding et al., 2023). During murine root morphogenesis, EDA1 has been mechanistically linked to direct modulation of Hertwig’s epithelial root sheath proliferation and planar orientation angles, which leads to enlarged pulp chambers, delayed furcation morphogenesis, and ultimately compromised root formation (Fons Romero et al., 2017).

*Eda* ^−/−^ mice exhibit compromised enamel organization, manifesting as hypoplastic, hypomineralized incisors with structural fragility (Yamada et al., 2020), possibly associated with attenuated NF-κB transcriptional activation in ameloblasts. This may result from mutant EDA1-mediated disruption of the EDA-EDAR-NF-κB axis, which directly compromises ameloblastic differentiation and enamel mineralization (Yamada et al., 2020). The EDA-NF-κB pathway regulates odontogenesis through crosstalk with canonical Wnt and BMP signaling cascades. Clinically, patients harboring dual mutations in *EDA* and *WNT10A* have exacerbated congenital TA phenotypes (Liu et al., 2024). However, the precise identity of downstream effector molecules regulated by the EDA-NF-κB axis during tooth morphogenesis remains unclear.

Transcriptomic profiling via RNA sequencing (RNA-seq) of dermal biopsies from male crossbred canines with ectodermal dysplasia has indicated crosstalks between EDA signaling and other pathways or other core molecules. Differentially expressed genes (DEGs) have been noted to be predominantly enriched in evolutionarily conserved pathways, including the Wnt/β-catenin, TNF superfamily, and TGF-β/BMP pathways, demonstrating that EDA pathway dysregulation induces destabilization of ectodermal homeostasis (Waluk et al., 2016).

In 2000, Nishikawa et al. investigated the localization of AP-1 family proteins in rat incisor ameloblasts via immunocytochemistry, detecting the presence of JUN, JUNB, JUND, FOS, FOSB, FRA-1, FRA-2 (Nishikawa, 2000); notably, only the FOS-specific antibody failed to exhibit staining. Subsequently, a 2002 study comparing the skin of wild-type, *Tabby*, and *Tabby* mice supplemented with the EDA1 isoform revealed that *Tabby* mice experienced significant downregulation of both the NF-κB signaling pathway and the JNK/FOS/JUN pathway and its target genes. The mechanisms through which EDA signaling coordinates transcriptional regulation of core downstream effectors and modulates cellular functionality during odontogenesis warrant further elucidation. Building on our previous findings that wild-type EDA1 promotes the proliferation of ameloblast-like cells via cell cycle regulation (Liu et al., 2021), the current study expands the investigation to additional cellular behaviors, specifically migration, apoptosis, and adhesion. To identify downstream differentially expressed genes (DEGs), for the first time, we performed comparative transcriptome sequencing on LS8 cells expressing wild-type EDA1 versus its pathogenic variants. These DEGs will be further validated through subsequent *in vivo* and *in vitro* experiments, thereby establishing a molecular foundation for exploring new therapeutic targets.

## Materials and methods

### Cells and groupings

LS8, a murine-derived ameloblast-like cell line, was kindly provided by Prof. Snead Malcolm (Center for Craniofacial Molecular Biology, Herman Ostrow School of Dentistry, University of Southern California, Los Angeles, CA, United States); these cells were continuously passaged in our laboratory. We constructed the eukaryotic expression vector pCMV-C-FLAG by inserting the coding sequence of either Wt or mutant EDA1 TNF structural domains. The experimental groups were designed as follows: Wt, NSTA-associated variant (EDA1-A259E) (NSTA), X-linked hypohidrotic ectodermal dysplasia-associated variant (EDA1-H252L) group (XLHED), and control group. All constructs were verified through sequencing and stored at −20 °C.

### Plasmid transformation, amplification, and extraction

Four recombinant plasmids were transformed into *Escherichia coli* BMXL1-Blue competent cells by using the heat-shock method. Plasmids were extracted using an EndoFree Plasmid Maxi Kit (Qiagen, Hilden, Germany) and sequenced. Verified plasmids were aliquoted and stored at −20 °C.

### LS8 cell culture and plasmid transfection

LS8 cells were routinely passaged and cultured in low-glucose Dulbecco’s modified Eagle’s medium (DMEM; Gibco, Thermo Fisher Scientific, Grand Island, NY, United States) supplemented with 10% fetal bovine serum and maintained at 37 °C in a humidified 5% CO_2_ incubator. For transfection experiments, the cells were seeded in six-well plates at 0.7 × 10^5^ cells/mL. Transfection was performed 20–24 h after seeding, when the cells reached 50%–60% confluency.

### Apoptosis detection

After 48 h of transfection, the culture medium and detached cells were centrifuged at 1,000 r/min at 4 °C for 5 min. The supernatant was discarded, and the cell pellet was washed once with ice-cold 1× phosphate-buffered saline (PBS). The cells were subsequently resuspended in 1 mL of 1× binding buffer.

For apoptosis detection, 100 μL of the cell suspension (about 1 × 10^5^ cells) was aliquoted into 2-mL microcentrifuge tubes. Next, 5 μL of the FITC–annexin V fluorescent probe and 5 μL of propidium iodide staining solution were added to each tube, and the cells were gently swirled, followed by incubation at 25 °C for 15 min in the dark. Next, 400 μL of 1× binding buffer was added to adjust the sample volume. Finally, flow cytometry (BD FACSCanto Ⅱ; BD Biosciences, Franklin Lakes, NJ, United States) was performed within 1 h after staining, with compensation controls included in each experiment.

### Cell scratching experiment

LS8 cells were transfected for 12 h and then subjected to the wound-healing assay. Three longitudinal scratches were created per well by using a sterile cell scraper, followed by a washing with PBS. Next, 2 mL serum-free DMEM was added, and the cells were imaged after 0, 6, 12, and 24 h under an inverted phase-contrast microscope. Wound areas were quantified using ImageJ, and migration rate (%) was calculated as [(initial area − timepoint area)/initial area] × 100.

### Cell adhesion and viability assay

We coated 96-well plates with 1 mg/mL Matrigel basement membrane matrix at 100 μL per well, followed by incubation at 37 °C for 2 h for polymerization. After the excess matrix was aspirated, the wells were washed with 1× PBS. Nonspecific binding sites were blocked with 1% bovine serum albumin at 100 μL per well at 37 °C for 1 h, followed by a washing with PBS. LS8 cells transfected for 48 h were seeded into precoated plates in triplicate; moreover, three replicate wells were included for each of the washed and unwashed groups. Cell viability was assessed using a cell counting kit (CCK) 8. In brief, cells per well were incubated with 100 μL of serum-free DMEM and 10 μL of CCK-8 reagent at 37 °C under 5% CO_2_ for 2 h in the dark. Absorbance was measured at 450 nm on a microplate reader. Finally, the adhesion rate was calculated as follows:

$$\text{Adhesion rate }\left(\%\right)=\frac{\left({\text{OD}}_{\text{Washed}}-ODBlank\right)}{\left(ODUnwashed-ODBlank\right)}\times 100\%$$

### RNA-seq

LS8 cells transfected with Wt EDA1 and its variants were seeded in a six-well plate and cultured for 48 h, with three biological replicates per group. Samples were sent to iGeneTech Bioscience Co., Ltd. (Beijing, China) for transcriptome sequencing. In brief, total RNA samples were first subjected to quality control. Libraries were then constructed and assessed for quality. The qualified libraries were sequenced on the Illumina NovaSeq 6000 platform to generate 150-bp paired-end reads. Finally, Illumina (or BGI) platform data were used for bioinformatic analysis. Differential expression analysis was performed using edgeR (https://bioconductor.org/packages/release/bioc/html/edgeR.html). Genes with |log_2_ (fold change)| >1 and adjusted *P* < 0.05 were defined as significantly differentially expressed. In cases where the number of DEGs was insufficient, a secondary screening threshold (nominal *P* < 0.05 and |log_2_ (fold change) | >1) was applied for exploratory analysis.

### Real-time quantitative polymerase chain reaction

We designed primers (Table 1) and sent them for synthesis by Sangyo Bioengineering (Shanghai, China). All procedures were performed on ice. RNA was extracted using an RNA extraction kit (Sangon Biotech, Shanghai, China), and genomic DNA was removed in a thermocycler (Bio-Rad Laboratories, Hercules, CA, United States). cDNA was synthesized through reverse transcription. Quantitative polymerase chain reaction (qPCR) was performed on a Biosystems 7500Fast Real-Time PCR System (Thermo Fisher Scientific, Waltham, MA, United States) with Takara TB Green Premix Ex TaqTM Ⅱ (Tli RNaseH Plus) (Takara Bio, Shiga, Japan) and primers for *Fos*, *Jun*, *Fosb*, and *Junb*, according to the manufacturer’s instructions.

**TABLE 1:** qPCR primers used here.

Gene	Forward (5′–3′)	Reverse (5′–3′)
*Fos*	GTG​GCC​TCC​CTG​GAT​TTG​A	CAC​GTT​GCT​GAT​GCT​CTT​GAC
*Jun*	GAA​GTG​ACG​GAC​CGT​TCT​ATG​ACT	GGAGGAACGAGGCGTTGA
*Fosb*	ACC​TGT​CTT​CGG​TGG​ACT​CCT​TC	AAG​ATC​CTG​GCT​GGT​TGT​GAT​TGC
*Junb*	CCT​GGA​CGA​CCT​GCA​CAA​GAT​G	GTA​ACT​GCT​GAG​GTT​GGT​GTA​GAC​G

### Immunoblotting

Culture medium was aspirated from each well of the six-well plates on ice, and the wells were gently washed with 1× PBS. Next, 190 μL of lysis buffer was added to each well, followed by incubation on ice for 40 min. Cells were subsequently scraped with a cell scraper, pipetted 5–10 times, and transferred to microcentrifuge tubes. After centrifugation at 14,000 r/min at 4 °C for 5 min, supernatants were collected. Protein concentration was quantified using the BCA Protein Assay Kit (Sangon Biotech, Shanghai, China), followed by electrophoresis and transfer onto polyvinylidene fluoride membranes. These membranes were incubated with anti-Flag antibodies (Abcam, Cambridge, United Kingdom) at 4 °C overnight. Next, they were treated with secondary antibodies on a shaker in the dark. Finally, the membranes were scanned using an Odyssey DLx Imaging System (LI-COR Biosciences, Lincoln, NE, United States), and the results were statistically analyzed.

### RNAScope analysis

Pregnant C57BL/6 and Tabby mice at various stages of early fetal development were selected for euthanasia under anesthesia (IACUC-Hebmu-2023042). The head tissue of the fetal mice was extracted and fixed in 4% paraformaldehyde at 4 °C for 24 h. The tissue was then embedded in the coronal position after fixation, and 4-μm-thick tissue sections were prepared for RNAscope analysis by using an RNAscope Multiplex Fluorescent Reagent Kit v2 (Advanced Cell Diagnostics, Newark, CA, United States). An RNAscope Z-type Target Probe (Advanced Cell Diagnostics) was then hybridized to the target RNA (NM_008036.2) for 2 h, and fluorescence was developed according to the specifications of TSA Vivid Fluorophore 520 (Bio-Techne, Minneapolis, MN, United States). The results were finally analyzed using 3DHISTECH.

### Statistical analysis

All experiments, including the migration, adhesion, cell apoptosis assay, qPCR, Western blot, RNA-seq, and RNAscope analyses, were performed using at least three biological replicates. Data were collected, analyzed, and confirmed across a minimum of three independent experimental repetitions. All quantitative data are presented as mean ± standard deviations. Student’s t*-*test was used to assess the statistical significance. *P* < 0.05 (*) and *P* < 0.01 (**) were considered to indicate statistical significance.

## Results

### EDA1 variants compromise early cellular migration but do not significantly affect apoptosis or adhesion

**FIGURE 1:**
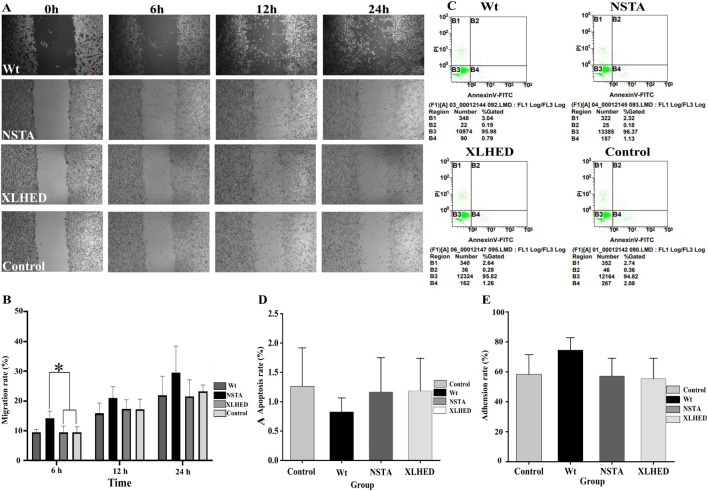
The Influence of ectodysplasin A1 (EDA1) on LS8 Cell behaviors **(A,B)** Effect of EDA1 variants on LS8 cell migration, quantified by the wound-healing assay. **(C,D)** Apoptotic response to EDA1 variants in LS8 cells, assessed by flow cytometry. **(E)** The effect of EDA1 variants on the adhesion of LS8 cells, measured by the cell counting kit 8 (CCK-8) assay. Data represent the mean ± standard deviation (*n* = 3). Statistical significance is denoted as *p < 0.05, **p < 0.01.

At 6 h, LS8 cells in the Wt group demonstrated a 32.1% enhancement in migratory capacity compared with both variant groups and the control group (*P* < 0.05; Figures 1A,B). However, no significant differences were observed in the migratory capacity between the variant and control groups at this timepoint (*P* > 0.05). At 12 and 24 h, migration rates in the Wt group remained elevated compared with the variant groups (19.0% and 23.7% increases, respectively); however, these differences were nonsignificant (*P* > 0.05). The Wt group exhibited a 35.4% reduction in the apoptosis rate compared with the control group; however, intergroup comparisons (Wt vs. variants vs. control) revealed no significant differences (*P* > 0.05; Figures 1C,D). Comparative analysis demonstrated a 24.9% increase in the cellular adhesion rate in the Wt group compared with the control group; however, intergroup comparisons (Wt vs. variants vs. control) revealed no significant differences (*P* > 0.05; Figure 1E).

### *Fos*, *Fosb*, *Jun*, and *Junb* are differentially expressed in cells carrying EDA1 variants

Genes were selected for downstream validation based on (i) statistical significance, prioritizing those with the largest fold changes and lowest p-values; (ii) biological relevance, emphasizing genes involved in GO and KEGG pathways associated with odontogenesis; and (iii) prior evidence from the literature implicating these genes in related developmental processes or diseases.

Comparative analysis revealed significant differential expression of *Fos*, *Fosb*, *Jun*, and *Junb* in the variant groups compared with the Wt group (*P* < 0.05). Transcriptomic profiling revealed extensive dysregulation of these genes across the variant groups; the NSTA group showed 71 upregulated and 79 downregulated genes, whereas the XLHED group exhibited 39 upregulated and 110 downregulated genes (Table 2).

**TABLE 2:** Number of DEGs in the control and variant groups compared with the Wt group.

Type	Control group	NSTA group	XLHED group
Upregulated	55	71	39
Downregulated	72	79	110

**FIGURE 2:**
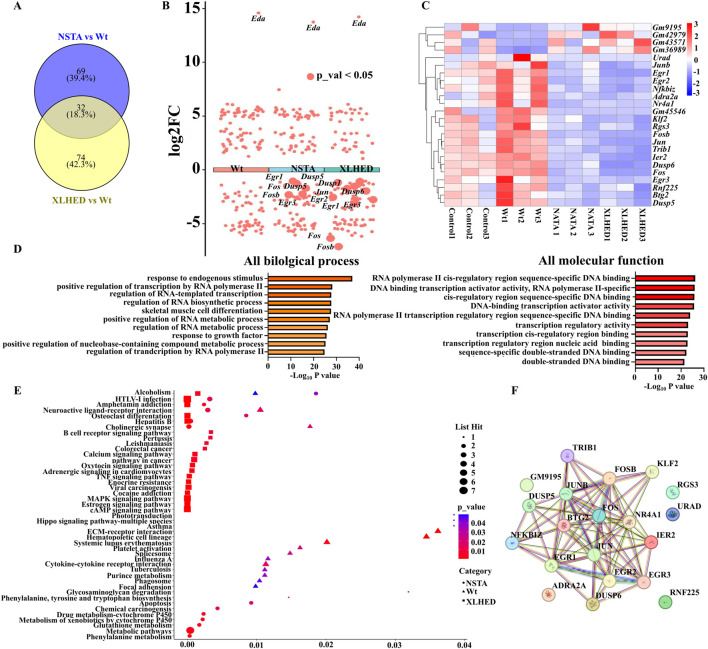
Transcriptomic analysis of differentially expressed genes (DEGs) in LS8 Cells **(A)** Venn diagram illustrating the overlap of DEGs identified from comparative RNA-seq analyses. **(B)** Volcano plot analysis of differential gene expression. **(C)** Heatmap visualization of the differentially expressed genes, highlighting clustering patterns across all experimental groups. **(D)** Gene Ontology (GO) enrichment analysis of the identified DEGs, focusing on biological processes and molecular functions. **(E)** Kyoto Encyclopedia of Genes and Genomes (KEGG) pathway enrichment analysis of DEGs. **(F)** Protein-protein interaction (PPI) network derived from the RNA-seq data, identifying key functional relationships. Data represent the mean ± standard deviation (*n* = 3).

Intersection analysis of DEGs in the NSTA, XLHED, and Wt groups revealed conserved pathogenic hub genes (Figure 2A). Subsequent volcano plot visualization and heatmap clustering confirmed this transcriptional repression pattern in the variant groups, particularly in AP-1 family members (*Fos*, *Fosb*, *Jun*, and *Junb*; Figures 2B,C). Transient EDA1 transfection significantly suppressed expression of the downstream EDA1-EDAR pathway effectors *Fos*, *Fosb*, *Jun*, and *Junb*. Table 3 presents the quantitative expression profiles of selected transcription factors.

**TABLE 3:** Expression levels of some of the most downregulated transcription factor genes in transiently transfected LS8 cells.

Gene	Wt group	Control group	NSTA group	XLHED group
*Fosb*	147.70	111.74	67.90	38.34
*Fos*	65.83	48.28	14.72	0.57
*Jun*	50.78	32.91	6.52	0.23
*Junb*	85.98	73.64	40.83	22.12

Comparative transcriptomic analysis revealed significant attenuation of core biological processes of the cells, including proliferation, migration, and differentiation, in the variant and Wt groups. Gene Ontology (GO) enrichment demonstrated downregulation of cAMP-responsive pathways (GO:0071377), MAPK signaling cascades (GO:0043408), and myeloid differentiation (GO:0030099) in the variant groups. Molecular function annotation revealed impaired MAP kinase phosphatase activity (tyrosine/serine/threonine-specific; GO:0004721-3), RNA polymerase II core promoter proximal DNA binding (GO:0000978), and transcription factor activity (GO:0003700; Figure 2D).

GO enrichment analysis demonstrated a strong association of these genes with the RNA biosynthetic process (GO:0032774) and DNA-templated transcription (GO:0006351; Figure 2D). Kyoto Encyclopedia of Genes and Genomes pathway enrichment analysis revealed significant enrichment of the TNF pathway (hsa04668), MAPK pathway (hsa04010), toll-like receptor pathway (hsa04620), osteoclast differentiation (hsa04380), and Wnt pathway (hsa04310) in the variant groups compared with the Wt group (Figure 2E).

We next constructed a protein–protein interaction network by using STRING DB (version 12.0) and identified nine hub genes with the highest node degrees: *Jun*, *Fos*, *Egr1*, *Dusp1*, *Fosb*, *Nr4a1*, *Btg2*, *Ier2*, and *Junb* (Table 4). The network revealed tight functional interconnectivity among AP-1 transcription factor family members (*Fos*, *Fosb*, *Jun*, and *Junb*) and immediate early response regulators (*Egr1* and *Ier2*; Figure 2F; Table 4).

**TABLE 4:** Degree in gene expression network.

Gene	Degree
*Jun*	50
*Fos*	48
*Egr1*	44
*Dusp1*	38
*Fosb*	32
*Nr4A1*	30
*Btg2*	30
*Ier2*	26
*Junb*	26

### EDA1 variants inhibit transcriptional activity of *Fosb* mRNA, impairing FOSB expression, in LS8 cells

**FIGURE 3:**
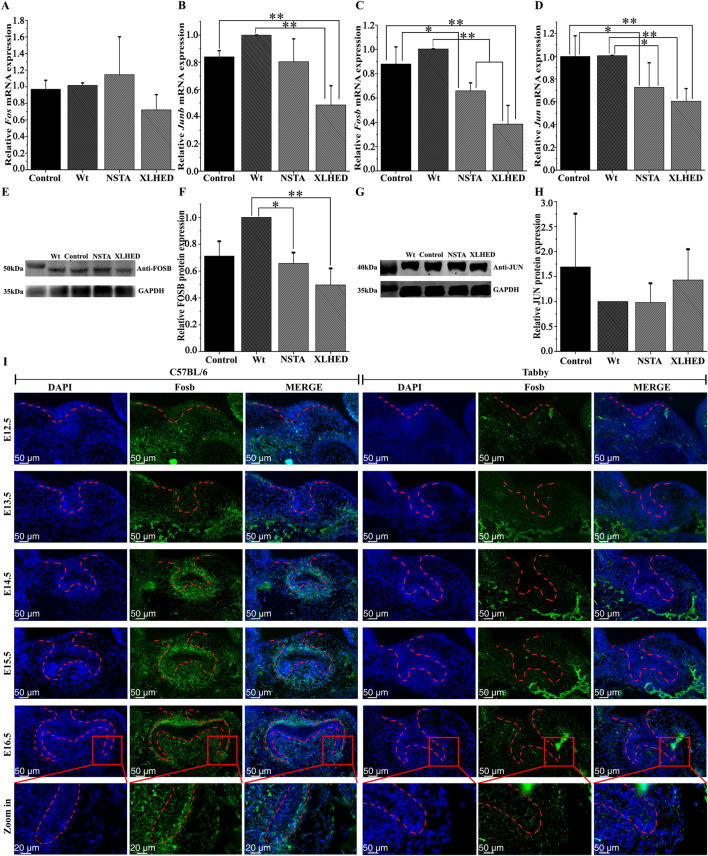
EDA1 variant effects on AP-1 gene and protein expression in LS8 Cells and *Fosb* localization *in vivo* **(A–D)** Relative mRNA expression levels in transfected LS8 cells, quantified by qPCR: **(A)** *Fos* mRNA expression; **(B)** *Junb* mRNA expression; **(C)** *Fosb* mRNA expression; **(D)** *Jun* mRNA expression. **(E–H)** Protein expression levels in transfected LS8 cells, analyzed by Immunoblotting: **(E)** Western Blot results for FOSB protein in LS8 cell lysate; **(F)** Relative expression of FOSB protein; **(G)** Western Blot results for JUN protein in LS8 cell lysate; **(H)** Relative expression of JUN protein. **(I)** RNAscope analysis showing distinct spatiotemporal expression patterns of *Fosb* mRNA in wild-type (C57BL/6) and *Tabby* mouse first molar germs during development. Data represent the mean ± standard deviations (*n* = 3). In the images from E12.5 to E16.5, the red dashed lines indicate the boundaries of the enamel organ, while in the zoom-in panels, the red dashed lines demarcate the cervical loop. Statistical significance is denoted as *p < 0.05, **p < 0.01.

qPCR for hub gene expression in LS8 cells demonstrated nonsignificant differences in *Fos* mRNA levels among the Wt, XLHED, NSTA, and control groups (*P* > 0.05; Figure 3A). Compared to the Wt group, the XLHED group demonstrated a 51.4% reduction in *Junb* mRNA expression (*P* < 0.01). The differences in *Junb* expression between the Wt, NSTA, and control groups were nonsignificant (*P* > 0.05). Compared with the control group, the XLHED group demonstrated a 35.4% reduction in *Junb* mRNA expression (*P* < 0.01). However, intergroup comparisons (control vs. NSTA vs. XLHED) demonstrated nonsignificant differences in *Junb* mRNA expression (*P* > 0.05; Figure 3B).

Compared with the Wt group, *Fosb* mRNA expression significantly decreased by 34.7% in the NSTA group (*P* < 0.01) and by 62% in the XLHED group (*P* < 0.01). Compared with the control group, *Fosb* mRNA expression significantly decreased by 22.4% in the NSTA group (*P* < 0.05) and by 49.7% in the XLHED group (*P* < 0.01; Figure 3C). qPCR analysis confirmed considerable suppression of *Fosb* mRNA expression in the variant groups.

Compared with the Wt group, *Jun* mRNA expression significantly decreased by 27.3% in the NSTA group (*P* < 0.05) and by 39.5% in the XLHED group (*P* < 0.01). Compared with the control group, *Jun* mRNA expression decreased by 27.1% in the NSTA group (*P* < 0.05) and by 39.3% in the XLHED group (*P* < 0.01; Figure 3D). Therefore, EDA1 variants may inhibit *Fosb* and *Jun* mRNA expression.

Next, we used immunoblotting to detect differences in the expression of the FOSB and JUN proteins in LS8 cells. Compared with the Wt group, FOSB protein expression was significantly decreased by 33.8% in the NSTA group (*P* < 0.05) and by 50.1% in the XLHED group (*P* < 0.01). However, no significant differences in FOSB protein levels were detected among the NSTA, XLHED, and control groups (Figures 3E,F). Similarly, JUN protein expression showed no statistical significance across all experimental conditions, including the Wt, NSTA, XLHED, and control groups (Figures 3G,H).

### *Fosb* mRNA expression is undetectable during early tooth germ development in Tabby mice

Distinct spatiotemporal patterns of *Fosb* mRNA expression were observed in the first molar germs of C57BL/6 mice. On embryonic day 12.5, RNAscope analysis revealed hyperplastic invagination of dental epithelium accompanied by differential expression profiles: proliferating epithelial cells demonstrated weaker *Fosb* mRNA expression, whereas the underlying condensed mesenchymal cells exhibited slightly stronger expression. By embryonic day 13.5, no specific *Fosb* mRNA expression was detected in either the dental epithelium or mesenchyme of the C57BL/6 mice. On embryonic day 14.5, strong *Fosb* mRNA expression was observed in the outer enamel epithelium, dental papillae, and dental follicles. During the bell stage on embryonic day 15.5, *Fosb* mRNA expression remained strong in the outer enamel epithelium, dental papillae, and dental follicles. By embryonic day 16.5, the primary domains of *Fosb* mRNA expression shifted to the outer enamel epithelium and cervical loops, with only weak residual expression in dental papillae. Notably, *Fosb* mRNA expression was entirely undetectable during all early developmental stages of tooth germ formation in *Tabby* mice, specifically including the bud, cap, and bell stages. Taken together, these results confirmed the essential regulatory role of EDA1 in *Fosb* transcriptional activation (Figure 3I).

## Discussion

Signaling molecules serve as pivotal regulators of organ morphogenesis, orchestrating essential cellular events, such as cytoskeletal remodeling, mitotic progression, and lineage specification, to maintain precise spatial and temporal coordination during tissue development (Gao et al., 2023). Tooth development depends on finely tuned epithelial–mesenchymal interactions and stage-specific activation of key signaling cascades, including Wnt, TGF-β, SHH, and FGF. Among these pathways, the EDA–EDAR axis plays a central role in initiating ectodermal organ formation by triggering NF-κB–mediated transcriptional programs that govern the sequential morphogenetic processes underlying odontogenesis (Mustonen et al., 2004; Gao et al., 2023; Shen et al., 2016; Mustonen et al., 2003).

During murine embryogenesis, *Eda* displays tightly controlled spatiotemporal expression, with weak epithelial signals first detectable at embryonic day 12.0 and markedly enhanced localization in the outer enamel epithelium by E14.0 (cap stage) (Tucker et al., 2000). Spatial mapping further reveals EDA1 enrichment within both the enamel knot, a cluster of non-dividing epithelial cells, and the adjacent odontogenic epithelium, where it contributes to tooth number specification and crown morphogenesis. The enamel knot functions as a transient but dynamic signaling center, whose emergence coincides with the onset of cusp formation (Tucker et al., 2000; Horakova et al., 2023; Jernvall et al., 1994). Similarly, *eda* ^−/−^ adult killifish exhibit shortened or twisted fin rays, malformed scales and teeth, and craniofacial deformities. A nonsense mutation in *eda1* (c.301G>T) induces panfin hypoplasia by disrupting the directed migration of Edar-expressing epithelial progenitors and reducing mesenchymal proliferation, thereby constraining fin elongation (Iida et al., 2014). In *Tabby* mice, the loss of EDA1 signaling results in defective cuspogenesis and enamel matrix hypoplasia (Pispa et al., 1999), accompanied by a range of incisor malformations, including hypoplastic, hypomineralized, fused, or missing incisors, with variable enamel defects (Grüneberg, 1965; Risnes et al., 2005; Sehic et al., 2009). Quantitative three-dimensional morphometric analyses further demonstrate progressive odontogenic impairment in these mutants, characterized by reduced buccolingual dimensions at E13.5, abnormal morphometric parameters at E14.5–15.5, and eventual loss of labiolingual asymmetry by E17.5 (Miard et al., 1999).

Organogenesis relies fundamentally on core cellular processes, proliferation, migration, apoptosis, and adhesion, that collectively orchestrate tissue formation, maintenance, and remodeling throughout development. Building on our previous findings that Wt EDA1 facilitates the G1/S-phase transition in LS8 cells, whereas the XLHED variant lose the ability (Liu et al., 2021), the present study was designed to further elucidate the downstream mechanisms governing these cellular behaviors. The morphogenesis of ectodermal organs requires tightly coordinated cellular events that determine tissue architecture and patterning. In tooth development, programmed cell death within the epithelial enamel knot drives the regression of this transient signaling center and coordinates crown morphogenesis, while apoptosis in secondary enamel knots helps define cusp boundaries and enamel fold patterning (Dutta et al., 2006). Beyond the enamel knot, apoptosis occurs in multiple dental cell populations, including ameloblasts, odontoblasts, and cells of the dental papilla and stellate reticulum, during critical stages of dentin formation (Matalova et al., 2004). These processes are governed by interconnected signaling networks, particularly NF-κB, whose activation is elevated during ectodermal appendage formation. Disruption of NF-κB signaling intensifies apoptosis and leads to morphological abnormalities across ectodermal derivatives, underscoring the importance of maintaining homeostatic balance during development. Previous studies have shown that EDA1 signaling modulates epithelial behavior, with stable expression enhancing extracellular matrix adhesion and promoting epithelial migration toward the basal lamina (Mustonen et al., 2004). Our current investigation provides new insights into how EDA1 coordinates multiple cellular behaviors in dental epithelium. Although Wt EDA1 suppressed apoptosis and enhanced adhesion in LS8 cells, effects that were abolished in the variants, the most robust and consistent influence across our models was on epithelial proliferation. The dental phenotypes observed in *Tabby* mice, including reduced tooth volume, simplified cusp patterns, and generalized hypoplasia, primarily reflect impaired proliferative expansion. Consistently, our prior work demonstrated that EDA1 promotes the G1/S-phase transition in both LS8 cells and hDPSCs (Ding et al., 2025). Within this regulatory framework, apoptosis, migration, and adhesion act as complementary mechanisms that refine and stabilize the fundamental proliferative program. The transient enhancement of migration (32.1% at 6 h) observed after *Eda* overexpression, together with its modulatory effects on apoptosis and adhesion, suggests that these processes play supportive yet essential roles in achieving precise morphogenetic outcomes. We therefore propose that EDA1-mediated proliferation serves as the primary driving force of dental epithelial morphogenesis, while the coordinated regulation of apoptosis, migration, and adhesion provides the necessary fine-tuning for proper tooth germ architecture and crown patterning.

The EDA1–NF-κB pathway interacts extensively with BMP and Wnt cascades during odontogenesis, thereby establishing the microenvironment necessary for tooth germ development (Pincha et al., 2022; Zhang et al., 2023; Wu et al., 2024). Transcriptomic profiling in the present study revealed that EDA1 variants disrupt cellular programs enriched in proliferation, migration, and differentiation. However, primary signaling effectors perturbed by EDA1 variants following homeostasis disruption remain unknown.

Comparative transcriptomic analyses of wild-type, *Tabby*, and *Eda*-transgenic *Tabby* mice have previously demonstrated that EDA1 deficiency suppresses both NF-κB and JNK/FOS/JUN signaling in skin epithelium (Cui et al., 2002). Our current data extend these findings to ameloblast-like cells, showing that EDA1 signaling functionally intersects with multiple pathways, including MAPK, Toll-like receptor, Wnt, and osteoclast differentiation pathways. Notably, *Fos*, *Fosb*, *Jun*, and *Junb* were consistently downregulated in EDA1 variants. This transcriptional pattern mirrors the known AP-1 expression profile in ameloblasts, where JUN, JUND and FRA-2 are strongly expressed in rat incisor ameloblast nuclei (Nishikawa, 2000). These results suggest that the EDA1–NF-κB axis contributes to dental epithelial homeostasis, at least in part, through coordinated regulation of AP-1 transcription factor components.

To validate the transcriptomic findings in dental epithelial cells, we confirmed a significant downregulation of *Fosb* and *Jun* mRNA in EDA1 variants compared with the wild-type and control groups. This transcriptional suppression was corroborated at the protein level, as immunoblotting revealed a marked reduction in FOSB expression, most pronounced in the XLHED group. Spatiotemporal localization analysis using RNAscope further provided *in vivo* evidence of *Fosb* regulation during tooth development. In C57BL/6 embryos, *Fosb* mRNA was predominantly expressed in the outer enamel epithelium at early stages of tooth germ development and gradually decreased in the dental papilla and follicle as maturation proceeded. Strikingly, *Fosb* expression was entirely absent throughout tooth germ development in *Tabby* mice, indicating that EDA1 deficiency abolishes *Fosb* activation within the dental epithelium as well as in the dental follicle and dental papilla, through a combination of direct epithelial effects and indirect epithelial-mesenchymal signaling.

As a member of the *Fos* gene family, *Fosb* is rapidly induced upon stimulation and promotes cellular proliferation. In late embryonic and neonatal mice, *Fosb* expression is highly enriched in developing bone and cartilage, and is also detected in whisker follicles, liver, and epidermal tissues. Consistent with its role in mineralized tissue formation, microarray and qPCR analyses of human dental follicle cells undergoing osteogenic differentiation have revealed upregulation of osteogenesis-associated genes, including *WNT2* and *FOSB* (Park et al., 2015). Alternative splicing of *Fosb* generates a truncated isoform, ΔFOSB (Krapacher et al., 2022), whose transgenic overexpression suppresses adipogenesis in mesenchymal stem cells but enhances osteogenesis. Although *Fosb*-deficient cells exhibit normal proliferation and S-phase entry following stimulation, they fail to upregulate other FOS family members and display reduced expression of two AP-1–related genes, underscoring the distinct transcriptional role of *Fosb* in regulating cell differentiation and tissue-specific gene expression.

Our *in vitro* analysis in mouse ameloblast-like cells demonstrated that EDA1 variants disrupt *Fosb* expression, thereby impairing enamel formation. Dynamic assessment of *Fosb* expression in C57BL/6 and *Tabby* mouse tooth germs revealed progressive *Fosb* mRNA upregulation from embryonic days 12.5–16.5 in C57BL/6 mice, whereas *Fosb* transcripts were completely absent in the tooth germs of *Tabby* mice. While our data highlight a strong correlation between FOSB and EDA1-mediated gene expression, its functional necessity and the precise mechanism require further validation. During tooth morphogenesis, *Eda* and *Edar* expression remains restricted to the epithelium (Laurikkala et al., 2001). The loss of *Fosb* expression corresponds closely with the characteristic *Tabby* dental phenotypes—namely small teeth, hypoplasia, hypomineralization, fusion or absence, and enamel defects. Notably, previous studies on dental pulp stem cells identified another AP-1 family member, FOS, as a critical regulatory factor (Ding et al., 2025), suggesting that distinct cell types within the dental organ may differentially engage AP-1 components in response to EDA signaling. Taken together, these findings imply that reduced *Fosb* expression downstream of the EDA signaling pathway may modulate key epithelial processes, such as migration, apoptosis, and adhesion.

## Conclusion

In conclusion, our findings demonstrate that Wt EDA1 promotes early-phase migration in LS8 cells, whereas pathogenic variants lose this pro-migratory capacity. Transcriptomic profiling and subsequent validation revealed that EDA1 variants markedly suppress *Fosb* transcription and translation, suggesting that FOSB acts as a candidate downstream effector of the EDA signaling pathway. The primary odontogenic mechanism regulated by the EDA signaling pathway likely involves epithelial regulation mediated through FOSB-depenment transcriptional control. These results provide important mechanistic insights into the molecular basis of tooth germ development and highlight potential therapeutic targets for *Eda*-associated developmental disorders.

## Data Availability

The datasets generated and/or analyzed during the current study are available from the corresponding author upon reasonable request.

## References

[B1] Ahmed H. A. El-Kamah G. Y. Rabie E. Mostafa M. I. Abouzaid M. R. Hassib N. F. (2021). Gene mutations of the three ectodysplasin pathway key players (EDA, EDAR, and EDARADD) account for more than 60% of Egyptian ectodermal dysplasia: a report of seven novel mutations. Genes. (Basel) 12 (9), 1389. 10.3390/genes12091389 34573371 PMC8468066

[B2] Borges G. H. Lins-Candeiro C. L. Henriques I. V. Brito Junior R. B. D. Pithon M. M. Paranhos L. R. (2025). Exploring the genetics, mechanisms, and therapeutic innovations in non-syndromic tooth agenesis. Morphologie 109 (364), 100941. 10.1016/j.morpho.2024.100941 39657464

[B3] Cai Z. Deng X. Jia J. Wang D. Yuan G. (2021). Ectodysplasin A/Ectodysplasin A receptor system and their roles in multiple diseases. Front. Physiol. 12, 788411. 10.3389/fphys.2021.788411 34938205 PMC8685516

[B4] Chow A. K. Low R. Yuan J. Yee K. K. Dhaliwal J. K. Govia S. (2024). Bioinformatics for dentistry: a secondary database for the genetics of tooth development. PLoS One 19 (6), e0303628. 10.1371/journal.pone.0303628 38843230 PMC11156362

[B5] Cui C. Durmowicz M. Tanaka T. S. Hartung A. J. Tezuka T. Hashimoto K. (2002). EDA targets revealed by skin gene expression profiles of wild-type, tabby and tabby EDA-A1 transgenic mice. Hum. Mol. Genet. 11 (15), 1763–1773. 10.1093/hmg/11.15.1763 12095918

[B6] Ding J. Liu H. Yu M. Liu Y. Han D. (2023). Measurement and analysis of The Crown conical degree of maxillary incisors in patients with congenital tooth agenesis caused by different gene mutations. Zhonghua Kou Qiang Yi Xue Za Zhi 58 (8), 821–828. 10.3760/cma.j.cn112144-20230328-00119 37550043

[B7] Ding Y. Lu G. Zhao Y. Zhang Y. Zhang J. Ma J. (2025). EDA1 variants inhibit the odontogenic differentiation and proliferation of human dental pulp stem cells. BMC Oral Health 25 (1), 358. 10.1186/s12903-025-05741-9 40057679 PMC11890513

[B8] Dutta J. Fan Y. Gupta N. Fan G. Gélinas C. (2006). Current insights into the regulation of programmed cell death by NF-κB. Oncogene 25 (51), 6800–6816. 10.1038/sj.onc.1209938 17072329

[B9] Fons Romero J. M. Star H. Lav R. Watkins S. Harrison M. Hovorakova M. (2017). The impact of the eda pathway on tooth root development. J. Dent. Res. 96 (11), 1290–1297. 10.1177/0022034517725692 28813629 PMC5613883

[B10] Gao Y. Jiang X. Wei Z. Long H. Lai W. (2023). The EDA/EDAR/NF-κB pathway in non-syndromic tooth agenesis: a genetic perspective. Front. Genet. 14, 1168538. 10.3389/fgene.2023.1168538 37077539 PMC10106650

[B11] Grüneberg H. (1965). Genes and genotypes affecting the teeth of the mouse. J. Embryol. Exp. Morphol. 14 (2), 137–159. 10.1242/dev.14.2.137 5893447

[B12] Hemeryck L. Hermans F. Chappell J. Kobayashi H. Lambrechts D. Lambrechts I. (2022). Organoids from human tooth showing epithelial stemness phenotype and differentiation potential. Cell. Mol. Life Sci. 79 (3), 153. 10.1007/s00018-022-04183-8 35217915 PMC8881251

[B13] Horakova L. Dalecka L. Zahradnicek O. Lochovska K. Lesot H. Peterkova R. (2023). Eda controls the size of the enamel knot during incisor development. Front. Physiol. 13, 1033130. 10.3389/fphys.2022.1033130 36699680 PMC9868551

[B14] Iida Y. Hibiya K. Inohaya K. Kudo A. (2014). Eda/edar signaling guides fin ray formation with preceding osteoblast differentiation, as revealed by analyses of the medaka all-fin less mutant afl. Dev. Dyn. 243 (6), 765–777. 10.1002/dvdy.24120 24585696

[B15] Jernvall J. Kettunen P. Karavanova I. Martin L. B. Thesleff I. (1994). Evidence for the role of the enamel knot as a control center in Mammalian tooth cusp formation: non-dividing cells express growth stimulating Fgf-4 gene. Int. J. Dev. Biol. 38 (3), 463–469. 7848830

[B16] Krapacher F. A. Fernández-Suárez D. Andersson A. Carrier-Ruiz A. Ibáñez C. F. (2022). Convergent dopamine and ALK4 signaling to PCBP1 controls FosB alternative splicing and cocaine behavioral sensitization. EMBO J. 41 (15), e110721. 10.15252/embj.2022110721 35730718 PMC10545536

[B17] Lan R. Wu Y. Dai Q. Wang F. (2022). Gene mutations and chromosomal abnormalities in syndromes with tooth agenesis. Oral Dis. 29 (6), 2401–2408. 10.1111/odi.14402 36219525

[B18] Laurikkala J. Mikkola M. Mustonen T. Aberg T. Koppinen P. Pispa J. (2001). TNF signaling *via* the ligand–receptor pair ectodysplasin and edar controls the function of epithelial signaling centers and is regulated by wnt and activin during tooth organogenesis. Dev. Biol. 229 (2), 443–455. 10.1006/dbio.2000.9955 11203701

[B19] Liu B. Kong X. Lu G. Zhang G. Jia X. Du Q. (2021). Effect of ectodysplasin-A1 on proliferation and cell cycle of ameloblast-like cell. Zhonghua Kou Qiang Yi Xue Za Zhi 56 (4), 349–354. 10.3760/cma.j.cn112144-20200816-00461 33832036

[B20] Liu Y. Sun J. Zhang C. Wu Y. Ma S. Li X. (2024). Compound heterozygous WNT10A missense variations exacerbated the tooth agenesis caused by hypohidrotic ectodermal dysplasia. BMC Oral Health 24 (1), 136. 10.1186/s12903-024-03888-5 38280992 PMC10822191

[B21] Matalova E. Witter K. Misek I. (2004). Apoptosis distribution in the first molar tooth germ of the field vole (*Microtus agrestis*). Tissue Cell. 36 (5), 361–367. 10.1016/j.tice.2004.06.006 15385152

[B22] Miard S. Peterková R. Vonesch J. L. Peterka M. Ruch J. V. Lesot H. (1999). Alterations in the incisor development in the tabby mouse. Int. J. Dev. Biol. 43 (6), 517–529. 10610025

[B23] Mustonen T. Pispa J. Mikkola M. L. Pummila M. Kangas A. T. Pakkasjärvi L. (2003). Stimulation of ectodermal organ development by Ectodysplasin-A1. Dev. Biol. 259 (1), 123–126. 10.1016/S0012-1606(03)00157-X 12812793

[B24] Mustonen T. Ilmonen M. Pummila M. Kangas A. T. Laurikkala J. Jaatinen R. (2004). Ectodysplasin A1 promotes placodal cell fate during early morphogenesis of ectodermal appendages. Development 131 (20), 4907–4919. 10.1242/dev.01377 15371307

[B25] Nishikawa S. (2000). Localization of transcription factor AP-1 family proteins in ameloblast nuclei of the rat incisor. J. Histochem Cytochem 48 (11), 1511–1520. 10.1177/002215540004801108 11036094

[B26] Park S. J. Bae H. S. Park J. C. (2015). Osteogenic differentiation and gene expression profile of human dental follicle cells induced by human dental pulp cells. J. Mol. Histol. 46 (1), 93–106. 10.1007/s10735-014-9604-1 25520056

[B27] Pincha N. Marangoni P. Haque A. Klein O. D. (2022). Parallels in signaling between development and regeneration in ectodermal organs. Curr. Top. Dev. Biol. 149, 373–419. 10.1016/bs.ctdb.2022.02.006 35606061 PMC10049776

[B28] Pispa J. Jung H. S. Jernvall J. Kettunen P. Mustonen T. Tabata M. J. (1999). Cusp patterning defect in tabby mouse teeth and its partial rescue by FGF. Dev. Biol. 216 (2), 521–534. 10.1006/dbio.1999.9514 10642790

[B29] Reinartz S. Weiß C. Heppelmann M. Hewicker-Trautwein M. Hellige M. Willen L. (2023). A missense mutation in the collagen triple helix of *EDA* is associated with X-Linked recessive hypohidrotic ectodermal dysplasia in fleckvieh cattle. Genes. 15 (1), 8. 10.3390/genes15010008 38275590 PMC10815684

[B30] Risnes S. Peterkova R. Lesot H. (2005). Distribution and structure of dental enamel in incisors of tabby mice. Arch. Oral Biol. 117 (6), 644–654. 10.1016/j.archoralbio.2004.11.003 15721148

[B31] Schneider H. Hadj-Rabia S. Faschingbauer F. Bodemer C. Grange D. K. Norton M. E. (2023). Protocol for the phase 2 EDELIFE trial investigating the efficacy and safety of intra-amniotic ER004 administration to Male subjects with X-linked hypohidrotic ectodermal dysplasia. Genes. (Basel) 14 (1), 153. 10.3390/genes14010153 36672894 PMC9858920

[B32] Sehic A. Peterkova R. Lesot H. Risnes S. (2009). Distribution and structure of the initial dental enamel formed in incisors of young wild-type and tabby mice. Eur. J. Oral Sci. 117 (6), 644–654. 10.1111/j.1600-0722.2009.00676.x 20121926

[B33] Shen W. Wang Y. Liu Y. Liu H. Zhao H. Zhang G. (2016). Functional study of ectodysplasin-a mutations causing non-syndromic tooth agenesis. PLoS One 11 (5), e0154884. 10.1371/journal.pone.0154884 27144394 PMC4856323

[B34] Tucker A. S. Headon D. J. Schneider P. Ferguson B. M. Overbeek P. Tschopp J. (2000). Edar eda interactions regulate enamel knot formation in tooth morphogenesis. Development 127 (21), 4691–4700. 10.1242/dev.127.21.4691 11023871

[B35] Waluk D. P. Zur G. Kaufmann R. Welle M. M. Jagannathan V. Drogemuller C. (2016). A splice defect in the EDA gene in dogs with an X-linked hypohidrotic ectodermal dysplasia (XLHED) phenotype. G3 (Bethesda) 6 (9), 2949–2954. 10.1534/g3.116.033225 27449516 PMC5015951

[B36] Wu Z. Wang Y. Han W. Yang K. Hai E. Ma R. (2020). EDA and EDAR expression at different stages of hair follicle development in cashmere goats and effects on expression of related genes. Arch. Anim. Breed. 63 (2), 461–470. 10.5194/aab-63-461-2020 33473371 PMC7810227

[B37] Wu Y. Lai L. Chen J. Li X. Hou J. (2024). Genotypic and phenotypic correlations in tooth agenesis: insights from *WNT10A* and *EDA* mutations in syndromic and non-syndromic forms. Hum. Genet. 143 (11), 1253–1264. 10.1007/s00439-024-02705-x 39320561

[B38] Yamada A. Kawasaki M. Miake Y. Yamada Y. Blackburn J. Kawasaki K. (2020). Overactivation of the NF-κB pathway impairs molar enamel formation. Oral Dis. 26 (7), 1513–1522. 10.1111/odi.13384 32369672 PMC8921976

[B39] Yu K. Huang C. Wan F. Jiang C. Chen J. Li X. (2023). Structural insights into pathogenic mechanism of hypohidrotic ectodermal dysplasia caused by ectodysplasin A variants. Nat. Commun. 14 (1), 767. 10.1038/s41467-023-36367-6 36765055 PMC9918506

[B40] Yu K. Sheng Y. Wang F. Yang S. Wan F. Lei M. (2024). Eight EDA mutations in Chinese patients with tooth agenesis and genotype-phenotype analysis. Oral Dis. 30 (7), 4598–4607. 10.1111/odi.14878 38287639

[B41] Zhang H. Gong X. Xu X. Wang X. Sun Y. (2023). Tooth number abnormality: from bench to bedside. Int. J. Oral Sci. 15 (1), 5. 10.1038/s41368-022-00208-x 36604408 PMC9816303

[B42] Zhao Z. Zhang T. Li T. Ye Y. Feng C. Wang H. (2023). A novel EDAR variant identified in non-syndromic tooth agenesis: insights from molecular dynamics. Arch. Oral Biol. 146, 105600. 10.1016/j.archoralbio.2022.105600 36470092

